# Investigation and validation of prognostic features associated with transient receptor potential channel genes in gastric cancer

**DOI:** 10.7717/peerj.21468

**Published:** 2026-08-11

**Authors:** Jinxia Zhao, Huihui Kang, Jiebin Pan, Pengfei Sun

**Affiliations:** 1 Department of Radiotherapye, The Second Hospital of Lanzhou University, Lanzhou, China; 2 Department of Oncology, The Second Hospital & Clinical Medical School, Lanzhou University, Lanzhou City, Gansu Province, China; 3 Department of General Surgery, The Second Hospital of Lanzhou University, Lanzhou, China

**Keywords:** Gastric carcinoma, Stomach adenocarcinoma, TCGA-STAD, Prognostic genes, Transient receptor potential channel

## Abstract

**Background:**

An increasing body of evidence suggests an association between transient receptor potential (TRP) channel s and cancer development; however, a systematic evaluation of the combined prognostic value of TRP channel-related genes (TRGs) in gastric cancer (GC) has not been conducted.

**Methods:**

Data were obtained from The Cancer Genome Atlas Stomach Adenocarcinoma, GSE84433, and TRGs. Prognostic genes related to TRP channels and GC were screened, and a risk model was constructed. Functional analysis, immune correlation analysis, and molecular regulatory network construction were performed to identify mechanisms associated with GC prognosis. Finally, the expression of prognostic genes was preliminarily assessed in clinical samples using reverse transcription quantitative polymerase chain reaction (RT-qPCR) for exploratory validation.

**Results:**

Seven genes associated with GC prognosis (5-hydroxytryptamine 2C receptor (*HTR2C*), *MAGEA11*, *MAGEA3*, aspartoacylase (*ASPA*), *GAD1*, Hedgehog-interacting protein (*HHIP*), and *AHNAK*) were identified. A risk model constructed from these genes demonstrated potential prognostic value for patient outcomes. A close association was observed between these genes and the differential abundance of immune cells. The strongest negative correlation was observed between AHNAK and T follicular helper cells. In addition, the prognostic genes were significantly negatively correlated with CD274, which is an immune checkpoint gene. Moreover, complex regulatory relationships among prognostic genes were observed in the regulatory network. Computational analysis predicted regulatory interactions, such as the regulation of *AHNAK* by EPB41L4A-AS1 and ERICD *via* hsa-miR-106a-5p. Finally, quantitative reverse transcription polymerase chain reaction (RT-qPCR) analysis revealed that the expression pattern of *MAGEA11* was consistent with that observed in public databases.

**Conclusions:**

Seven prognostic genes (*HTR2C*, *MAGEA11*, *MAGEA3*, *ASPA*, *GAD1*, *HHIP*, and *AHNAK*) related to TRP channels were identified in GC. These findings provide insight into potential prognostic mechanisms and a basis for further studies on their roles in GC biology.

## Introduction

Gastric cancer (GC) is the most common malignant tumor of the digestive system. In 2024, there will be an estimated 26,8900,000 new cases of GC and approximately 10,880,000 deaths worldwide (Siegel, Giaquinto & Jemal, 2024). With the rapid development of molecular biology research, GC treatment methods have continuously improved, including the introduction of targeted therapy and immunotherapy (Cai et al., 2022), providing new therapeutic options for patients with advanced GC. Although these treatments prolong survival in some patients, the prognosis for advanced GC remains poor (Zang et al., 2019). Therefore, there is a need to identify molecular markers that accurately predict the prognosis of patients with GC.

Signal transduction in cells is mediated through membrane-bound receptor proteins, of which ion channels play an important role in the real-time sensing of intracellular and extracellular signals and the regulation of adaptive cellular responses. Transient receptor potential (TRP) channels represent a diverse family of non-selective cation channels that regulate intracellular calcium homeostasis and participate in physiological and pathological processes, including cancer progression (He et al., 2022a; Marini et al., 2023). The TRP channel family contains multiple subtypes with tissue-specific expression patterns, including TRPV (vanilloid), TRPA (ankyrin), and TRPM (melastatin). For example, TRPV1 exhibits prominent expression in neurons and tumor cells, whereas TRPM8 shows significant upregulation in prostate cancer (PCa) (Murugan, Genautis & Voutsadakis, 2025; Yan et al., 2024). In the tumor microenvironment, dysregulated TRP channel expression across malignancies has been observed. TRPV6, TRPA1, and TRPM8 expression levels correlate with advanced tumor stages and invasiveness in pancreatic adenocarcinoma, hepatocellular carcinoma (HCC), and breast cancer (Chelaru et al., 2022; Wu et al., 2023; Zhang et al., 2022). Mechanistically, TRP channels orchestrate Ca^2^^+^-mediated signaling cascades that modulate tumor cell proliferation, migration, apoptosis, and immune evasion (Ferreira & Britto, 2023; Jiang et al., 2024), thus exhibiting dual regulatory roles in carcinogenesis (Ren et al., 2022). TRP channel-related genes (TRGs) are considered promising prognostic biomarkers for many cancer types (Guan et al., 2024; Shi et al., 2022). Recent studies have provided further evidence for the significance of TRP channels in GC. TRPV1 undergoes dynamic regulation during the development of GC, exhibiting markedly different expression profiles in cancerous tissues and precancerous lesions (Groen et al., 2024). Based on a review of TRPC6, TRPM2, TRPM7, and TRPV6 (Sterea, Egom & El Hiani, 2019), the role of TRP signaling in GC has been further elucidated, and the clinical relevance of TRPV2 has been established (Kato et al., 2022). Nevertheless, the prognostic significance and therapeutic implications of TRP channels in GC remain unclear, which warrants further investigation into their potential as molecular targets for GC management and outcome prediction.

In this study, the expression and prognostic value of TRP channels in patients with GC were analyzed using the Gene Expression Omnibus (GEO) and University of California, Santa Cruz (UCSC) Xena databases. The mechanisms associated with these genes may be involved in the occurrence or prognosis of GC. This study aims to enhance our understanding of the role of TRP channels in GC prognosis and to provide hypotheses for future functional studies. The results may ultimately lead to the development of novel biomarkers or therapeutic strategies for GC.

## Materials and Methods

### Data source

The transcriptomic data and corresponding clinical information, including sex, age at diagnosis, vital status, tumor stage, and survival time, were obtained from the UCSC Xena database (https://xena.ucsc.edu/). The raw count data were downloaded in HTSeq-Counts format for The Cancer Genome Atlas Stomach Adenocarcinoma (TCGA-STAD) project. The original dataset consisted of 375 primary GC tumor samples and 32 normal gastric tissue samples. After excluding samples with incomplete survival information, 348 GC tumor samples and 31 normal tissue samples were included for subsequent analysis. To ensure the data format strictly met the requirements of DESeq2, we used the round() function to round the count data to integers before the analysis. The GSE84433 dataset was downloaded from the GEO database (GPL6947 platform), which provides gene expression profiles and clinical information for 357 GC samples. After obtaining the data using the GEOquery package, the microarray data were processed as follows: (1) expression values were log(x + 1)-transformed, (2) probe IDs were converted to gene symbols, (3) the avereps function was applied to calculate the average expression value for genes corresponding to multiple probes, and (4) probes lacking corresponding gene symbols or containing “///” were removed. An expression matrix of 17,969 genes was ultimately obtained for subsequent analysis. A total of 120 TRGs were retrieved from the Molecular Signatures Database and the Kyoto Encyclopedia of Genes and Genomes (KEGG) database. The data were assessed on 26/01/2024.

### Differential expression analysis

Within the TCGA-STAD dataset, differential expression analysis between tumor and normal cohorts was performed using the DESeq2 package to identify differentially expressed genes (DEGs) with a threshold set at |log_2_FoldChange (FC)|> 1 and adjusted *p*-value (padj) < 0.05. DESeq2 performs differential expression analysis based on the negative binomial distribution model. First, the median-of-ratios method is used to estimate sample size factors for normalizing differences in sequencing depth and library composition. Subsequently, an empirical Bayes approach is used to robustly estimate gene-wise dispersion, shrinking the dispersion estimates toward the mean-dispersion trend line to stabilize estimation for low-expression genes. A generalized linear model is then fitted, and the Wald test is used to assess the significance of differences between groups. This algorithm demonstrates robust performance when handling imbalanced sample sizes.

### Weighted gene co-expression network analysis (WGCNA)

The WGCNA package was used to identify modules related to sample traits (tumor *vs.* normal). Hierarchical cluster analysis was carried out to detect and remove anomalous samples. A suitable soft threshold (β) was selected by ensuring a high *R*^2^ (>0.85) and low connectivity close to 0. Subsequently, a scale-free network was built using the chosen soft threshold, which resulted in the division of all genes into distinct modules represented by different colors (minModuleSize = 100 and mergeCutHeight = 0.15). Correlation coefficients between the co-expression modules and sample traits were computed, and the module exhibiting the highest correlation (|*r*| > 0.3, *p* < 0.05) was designated the key module.

### Procurement of candidate genes

Within the TCGA-STAD dataset, the single-sample Gene Set Enrichment Analysis (ssGSEA) algorithm was used to calculate TRP channel-related ssGSEA scores for all samples using 120 TRGs. The differential distribution of ssGSEA scores between the tumor and normal samples was analyzed. All specimens were divided into high- and low-ssGSEA score groups based on the median ssGSEA score as the threshold. Differential expression analysis was performed using the same thresholds and software packages as described above. The intersection of the DEGs from the differential expression analysis (tumor *vs* control, high *vs* low) and module genes from WGCNA was determined using ggVennDiagram, which resulted in a list of candidate genes.

### Functional analysis

Gene ontology (GO) and Kyoto Encyclopedia of Genes and Genomes (KEGG) functional enrichment analyses of the candidate genes were conducted using the clusterProfiler software package (padj < 0.05, Benjamini–Hochberg correction). Protein–protein interaction (PPI) relationships for the candidate genes were obtained using the Search Tool for the Retrieval of Interacting Genes/Proteins (STRING) database with a score above 0.15 (Li et al., 2025a). In the STRING database, a higher interaction score indicates greater reliability of the interaction. A low confidence threshold of 0.15 was used to include as many potential interactions as possible, thus broadening the screening scope and avoiding omissions.

### Build and validate risk models

GC specimens from the TCGA-STAD were partitioned into a training set (*N* = 244) and a validation set (*N* = 104) at a ratio of 7:3. The GSE84433 dataset was used as an external validation subset to construct and validate the risk model. Prognostic genes were identified by a univariate Cox regression analysis (*p*-value < 0.1) and evaluation of the Proportional Hazards (PH) hypothesis test (*p*-value > 0.05). Subsequently, the glmnet package was applied for least absolute shrinkage and selection operator (LASSO) regression analysis to select the prognostic genes for the risk model (family = Cox). Patients were stratified into a high-risk group (HRG) and a low-risk group (LRG) based on the optimal threshold for the classification of risk scores (risk score = I = 1ncoef(genei) ∗expr(genei)).

Kaplan–Meier (K–M) survival curves were generated for the two risk cohorts using the survminer package. For 1-, 3-, and 5-year survival estimations, the timeROC package was used to construct receiver operating characteristic (ROC) curves to assess the predictive accuracy of the model. Finally, its effectiveness and applicability were validated using the internal and external validation sets.

### Independent prognostic analysis

Using the TCGA-STAD dataset, clinical pathological features (including age, sex, tumor stage, T stage, N stage, M stage, and histological subtype) were integrated with the risk score to construct a risk model. Univariate Cox regression analysis, the PH hypothesis test (*p* > 0.05), and multivariate Cox regression analysis were performed to identify independent prognostic factors. Based on these independent prognostic factors, a nomogram was constructed using the rms package, and a calibration curve was plotted to evaluate the predictive accuracy of the model. A calibration curve slope closer to 1 indicates higher predictive accuracy.

### GSEA

Within the training subset, the variance in expression levels was examined between the two risk groups. The log_2_FC values were ranked in descending order and analyzed by GSEA using clusterProfiler for “c2.kegg.v7.4.symbols” data (padj < 0.05).

### Immunoinfiltration analysis

The compositional differences in immune cell infiltrates were determined within the training samples using CIBERSORT. Samples exhibiting significant differences were selected for subsequent analysis based on outcomes, and inter-cohort differences were compared (*p* < 0.05). The differences in the expression levels of eight conventional immune checkpoints between the two risk cohorts in the training set were analyzed (*p*-value <0.05). Spearman correlation analysis was conducted to determine the association among the prognostic genes, distinct immune cells, differential immune checkpoints, and risk score (|*r*|> 0.3, *p*-value < 0.05).

### Construction of regulatory networks

Based on the prognostic genes, microRNAs (miRNAs) were predicted using the miRDB database (http://mirdb.org/; target score ≥ 85) and the MicroT-CDS database (http://crdd.osdd.net/raghava/microt-cds/; miTG score ≥ 0.9). The intersection was obtained to identify the shared miRNAs. Subsequently, long non-coding RNAs (lncRNAs) were predicted using the miRNet (http://crdd.osdd.net/raghava/microt-cds/) and Starbase databases (http://starbase.sysu.edu.cn/; geneType = lincRNA and clipExpNum ≥ 5), and the overlap was obtained to identify shared miRNA–lncRNA relationships. Transcription factors (TFs) of the prognostic genes were predicted using the NetworkAnalyst database (https://www.networkanalyst.ca/) to identify potential upstream regulatory mechanisms of the prognostic genes. Finally, the mRNA–miRNA–lncRNA and TF–mRNA networks were visualized using Cytoscape.

### Reverse transcription quantitative polymerase chain reaction (RT-qPCR)

To validate the expression profiles of the prognostic genes in clinical samples, RT-qPCR was conducted on five paired GC and adjacent normal tissues collected from patients at the Second Hospital of Lanzhou University on 8/9/2025. Because of the limited sample size, the analysis was intended as an exploratory validation to complement the bioinformatics findings. This study was approved by the Institutional Medical Ethics Committee of the Second Hospital of Lanzhou University (approval no. 2025A-744). The patients signed written informed consent forms, and the study complied with local laws, regulations, and institutional requirements. Total RNA was isolated from each sample using TRIzol reagent (Ambion, USA), and reverse transcription was performed using the SureScript First Strand cDNA Synthesis Kit (Wuhan Saibo Biotechnology Co., Ltd.). RT-qPCR experiments were carried out using 2 × Universal Blue SYBR Green qPCR premix (Wuhan Saibo Biotechnology Co., Ltd.), with *GAPDH* serving as the housekeeping gene (Birerdinc et al., 2012). The PCR primer sequences are listed in Table S1. Each group consisted of five biological replicates, and three technical replicates were run for each sample. The average Ct value of the three technical replicates was used for subsequent analysis. Relative gene expression was calculated using the 2^−ΔΔCt^ method, and statistical analysis and visualization were conducted using GraphPad Prism (v 10.0).

### Statistical analysis

Data processing and analysis were conducted using R software (v 4.3.1) and GraphPad Prism 10.0. The Shapiro–Wilk test was used to assess data normality. For the bioinformatics data, because most datasets did not follow a normal distribution, the Wilcoxon signed-rank test was used for intergroup comparisons. Correlation analysis was performed using Spearman’s rank correlation coefficient, with |*r*| > 0.3 and *p* < 0.05 as the screening criteria. To control the false positive rate during multiple comparisons, the Benjamini–Hochberg method was used to calculate padj, with padj < 0.05 as the threshold for identifying DEGs. For RT-qPCR validation in five paired samples, genes conforming to normality were analyzed using paired *t*-tests, whereas those not conforming to normality were analyzed using paired Wilcoxon signed-rank tests. A *p*-value < 0.05 was considered statistically significant.

## Results

### A total of 1,997 key module genes were identified

**Figure 1:**
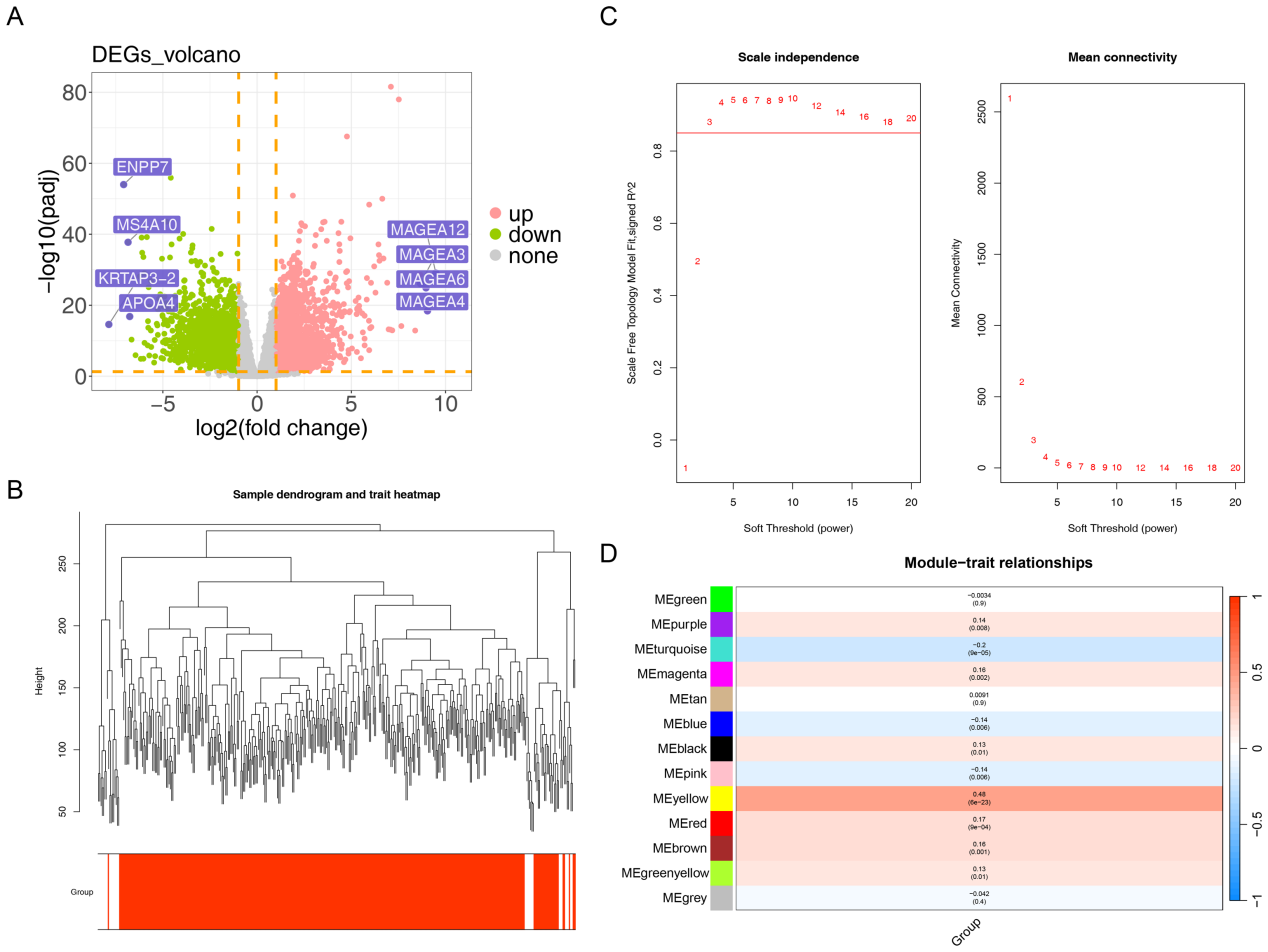
Identification of differentially expressed genes and key modules in gastric cancer. (A) Volcano plot showing differentially expressed genes between tumor and normal samples in the TCGA-STAD dataset. Red and green dots represent significantly upregulated and downregulated genes, respectively (|log_2_ FC| > 1, padj < 0.05). (B) Clustering dendrogram showing no obvious outliers. (C) Soft thresholding power selection (*β* = 5) for scale-free network topology (*R*^2^ > 0.85). (D) Heatmap showing the correlations between the modules and sample traits (tumor *vs.* normal). The yellow module exhibited the highest correlation (*r* = 0.48, *p* < 0.05) and was selected as the key module.

Within the TCGA-STAD dataset, 2,138 upregulated and 2,369 downregulated DEGs were identified, totaling 4,507 genes (Figs. 1A, S1A). The sample cohort in the TCGA-STAD dataset was used to select modules that were significantly associated with the trait. Initially, hierarchical clustering analysis revealed no outlier samples (Fig. 1B). A soft threshold of 5, with an *R*^2^ value greater than 0.85 and a near-0 connectivity, was selected (Fig. 1C). Twelve co-expression modules were identified, with the yellow module exhibiting the strongest association with the trait (*r* = 0.48, *p* < 0.05). It was defined as the key module and contained 1,997 key module genes (Figs. 1D, S1B).

### Candidate genes were enriched in multiple signaling pathways

**Figure 2:**
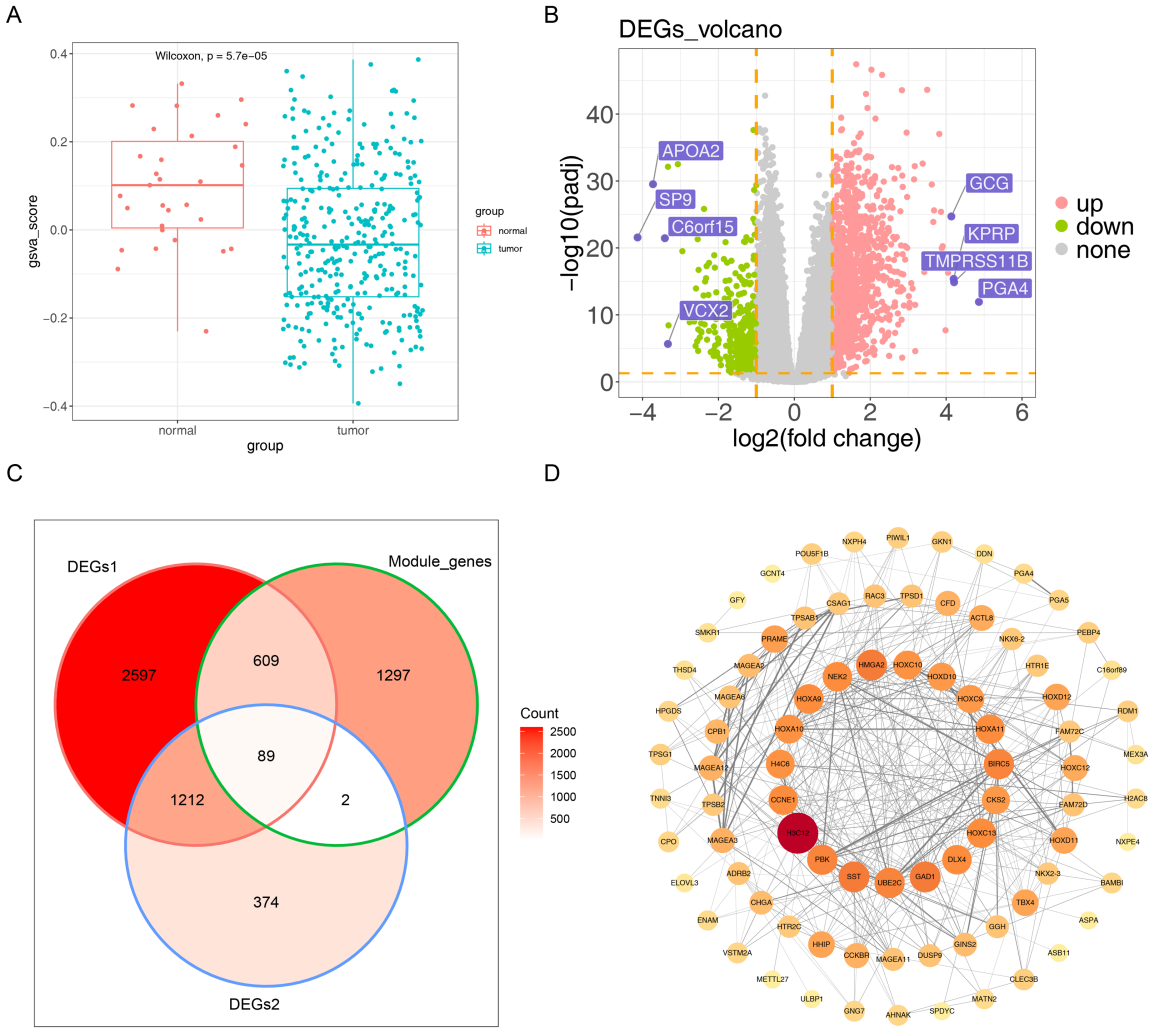
Enrichment and interaction analysis of candidate TRP channel-related genes. (A) Box plot comparing TRP channel-related ssGSEA scores between the tumor and normal samples. *p* < 0.001, Wilcoxon rank-sum test. (B) Volcano plot for the differentially expressed genes between high- and low-ssGSEA score groups. Pink: upregulated (*n* = 1,299); green: downregulated (*n* = 378); gray: not significant (|log_2_ FC| > 1, padj < 0.05). (C) Venn diagram showing the intersection of DEGs (tumor *vs.* normal), DEGs (high- *vs.* low-ssGSEA score), and key module genes, which yielded 89 candidate genes. (D) Protein –protein interaction network of candidate genes constructed using the STRING database (confidence score > 0.15). H3C12 was identified as a hub gene.

In the TCGA-STAD dataset, TRP channel-related ssGSEA scores were calculated for all samples and revealed significant differences in ssGSEA scores between tumor and normal samples, with lower scores in the tumor cohort (Fig. 2A). Using the median ssGSEA score as a criterion, all samples were segregated based on high- and low-ssGSEA scores, enabling the examination of differential expression profiles. A total of 1,677 DEGs were identified between the two cohorts, which included 1,299 upregulated and 378 downregulated DEGs (Figs. 2B, S1C). The intersection of the DEGs between the cohorts (tumor *vs* control, high *vs* low) and key module genes revealed 89 candidate genes (Fig. 2C). A GO analysis indicated that the candidate genes were primarily enriched in limb development, protein-coupled amine receptor activity, and nuclear chromosome functions. A KEGG analysis (Fig. S1D) revealed that these genes were predominantly involved in pathways, such as the cAMP signaling pathway and serotonergic synapse (Fig. S1E)). Finally, in the PPI network, H3C12 was identified as a core gene and interacted with proteins encoded by other candidate genes (Fig. 2D).

### Prognostic model demonstrated potential value in predicting outcomes for GC

**Figure 3:**
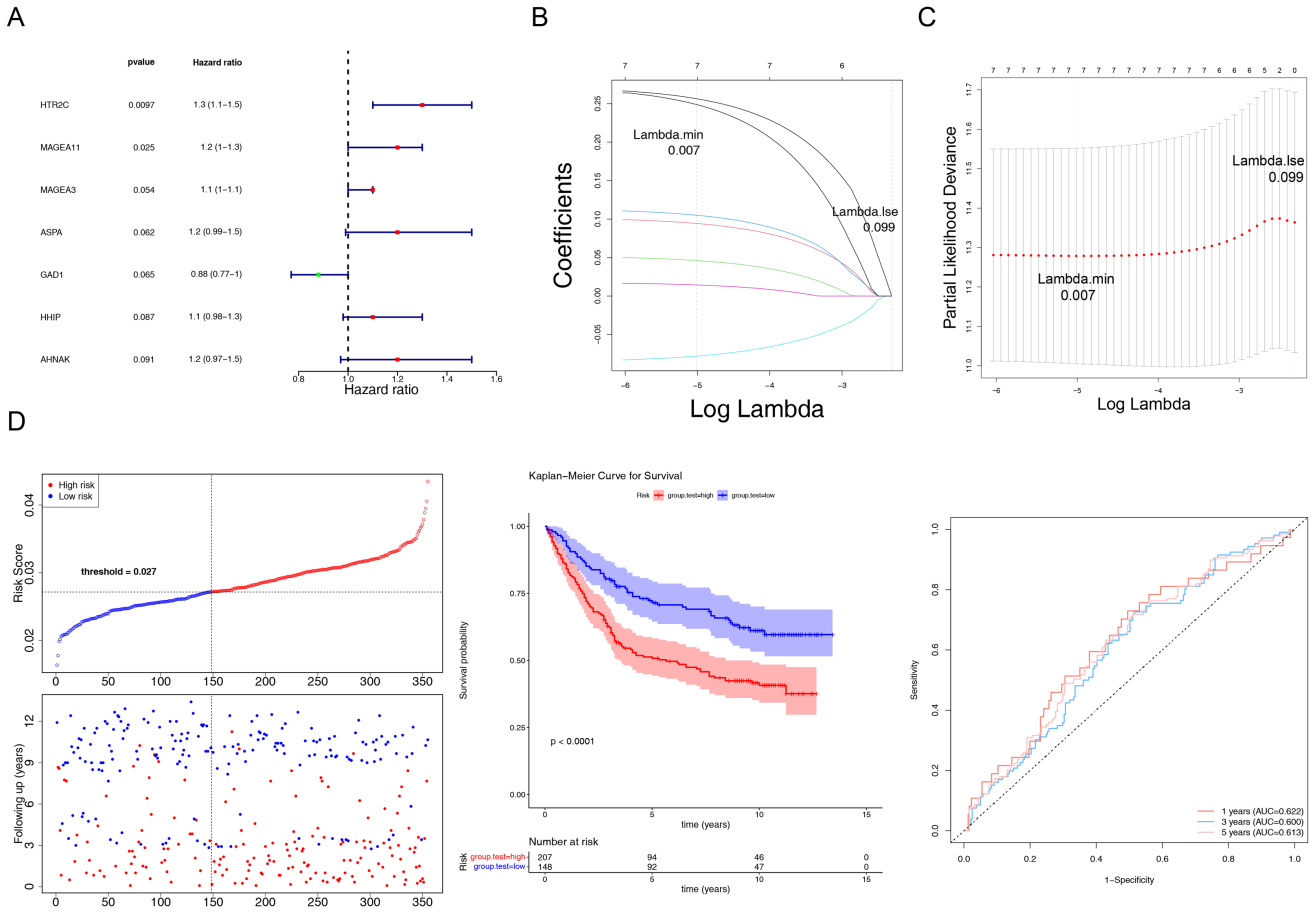
Construction and validation of the TRP channel-related prognostic model. (A) Forest plot of univariate Cox regression analysis identifying seven prognosis-related genes, including *HTR2C*, *MAGEA11*, *MAGEA3*, *ASPA*, *GAD1*, *HHIP*, and *AHNAK*, in the TCGA-STAD training set (*p* < 0.1). (B, C) LASSO regression coefficient profiles of the seven genes. Cross-validation for tuning parameter selection in LASSO regression (lambda.min = 0.007). (D) External validation using the GSE84433 dataset (*n* = 357). (D-1) Risk score distribution and survival status. (D-2) Kaplan–Meier survival curves (log-rank test, *p* < 0.0001). (D-3) Time-dependent ROC curves for predicting 1-, 3-, and 5-year survival.

The TCGA-STAD dataset was divided into two subsets using a randomized 7:3 ratio, with one subset of 244 samples designated as the training set and the remaining 104 samples as the internal validation set. In the training set, seven prognostic genes (5-hydroxytryptamine 2C receptor (*HTR2C*), *MAGEA11*, *MAGEA3*, aspartoacylase (*ASPA*), *GAD1*, Hedgehog-interacting protein (*HHIP*), and *AHNAK*) were identified by univariate Cox regression analysis and satisfied the PH for further LASSO analysis (Fig. 3A, Table 1). When the lambda min was set to 0.007, seven prognostic genes were selected (*HTR2C*, *MAGEA11*, *MAGEA3*, *ASPA*, *GAD1*, *HHIP*, and *AHNAK*; Figs. 3B, 3C). In the training set, patient risk scores were determined by integrating gene expression levels with their respective risk coefficients (Table 2). The death rate in the HRG increased with higher scores (Fig. S2A). K–M curves (*p* < 0.0001) showed significant survival differences between the HRG and LRG, indicating that survival among the HRG was significantly lower (Fig. S2A). In addition, the area under the curve (AUC) values for 1-, 3-, and 5-year survival exceeded 0.6, suggesting that the model exhibits moderate predictive capacity for patient outcomes at different time points (Fig. S2A). The risk model demonstrated consistent prognostic stratification capability in the internal and external validation cohorts, further supporting its potential generalizability (Figs. S2B, 3D).

**Table 1:** Results of univariate Cox regression analysis.

id	*z*	HR	HR.95L	HR.95H	pvalue	PH
HTR2C	2.58482	1.28189	1.06187	1.54749	0.00974	0.72596
MAGEA11	2.23788	1.15287	1.01782	1.30583	0.02523	0.15319
MAGEA3	1.92332	1.07026	0.99871	1.14695	0.05444	0.16512
ASPA	1.86427	1.2013	0.99063	1.45676	0.06228	0.68784
GAD1	−1.84228	0.88142	0.77066	1.0081	0.06543	0.25564
HHIP	1.71076	1.11283	0.98455	1.25782	0.08713	0.14028
AHNAK	1.68885	1.19134	0.97229	1.45974	0.09125	0.56674

**Table 2:** Differences in risk scores among different clinical characteristics.

	**coef**	**exp(coef)**	**se(coef)**	***z***	***p***
***HTR2C***	0.27252	1.31327	0.13202	2.064	0.039
***MAGEA11***	0.10216	1.10756	0.07837	1.304	0.1924
***MAGEA3***	0.05242	1.05382	0.04915	1.067	0.2861
***ASPA***	0.11381	1.12054	0.12199	0.933	0.3508
***GAD1***	−0.08566	0.91791	0.08449	−1.014	0.3107
***HHIP***	0.01801	1.01817	0.07296	0.247	0.805
***AHNAK***	0.27403	1.31525	0.13305	2.06	0.0394

### Risk score as an independent prognostic factor

**Figure 4:**
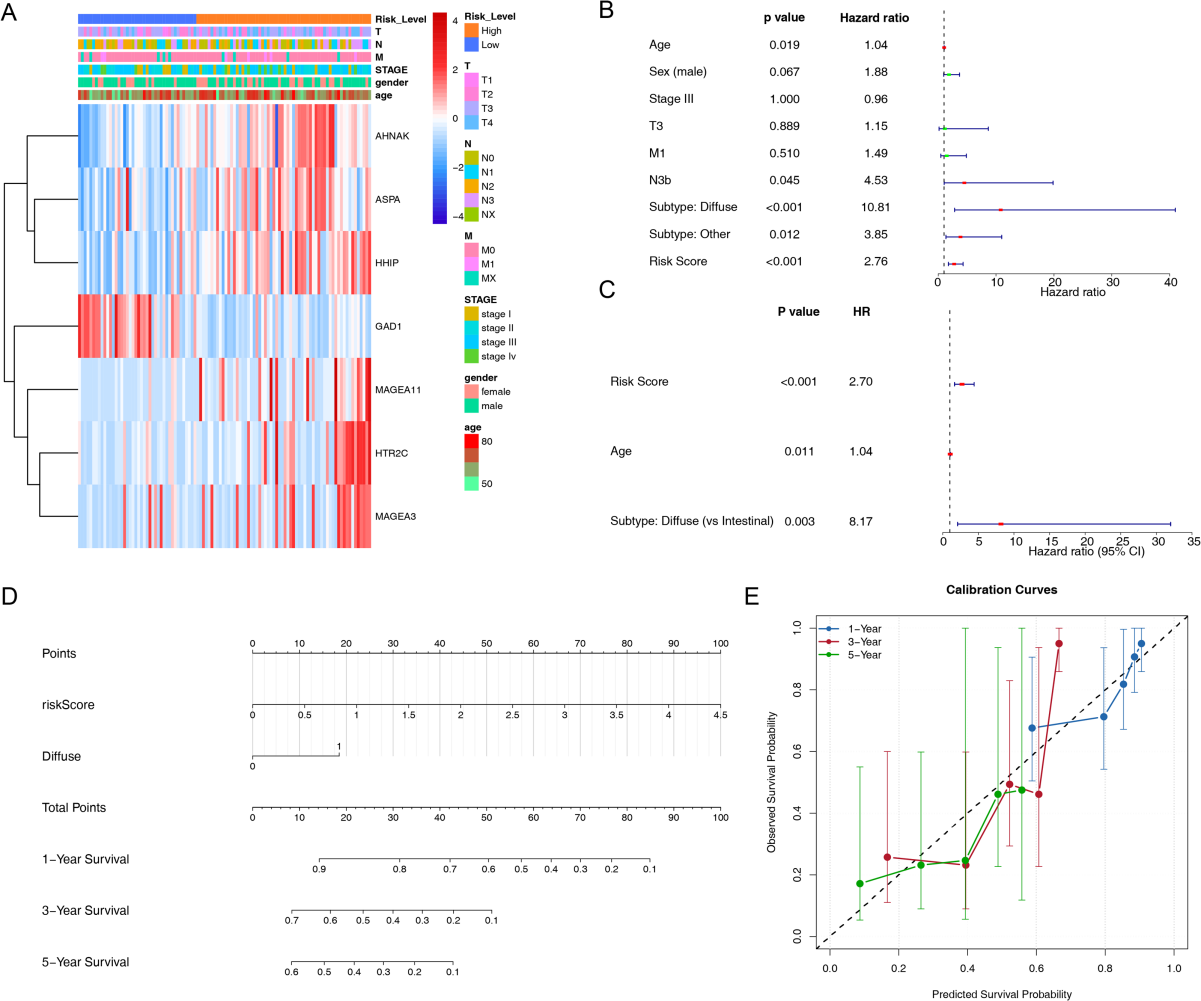
Independent prognostic value of the risk score. (A) Analysis of prognostic gene expression for various clinicopathological factors. (B) Univariate Cox regression analyses of risk score and clinical features. (C) M ultivariate Cox regression analyses of risk score and clinical features. Risk score and histological subtype were identified as independent prognostic factors (*p* < 0.01). (D) Nomogram integrating risk score to predict 1-, 3 -, and 5 -year overall survival. (E) Calibration curves for the nomogram-predicted *vs.* observed survival probabilities at 1, 3 , and 5 years. A slope close to the diagonal line indicates good predictive accuracy.

Analysis of prognostic gene expression across different clinicopathological factors (age, gender, stage, T, N, and M) revealed that *GAD1* expression was significantly higher in the LRG (Fig. 4A). Independent prognostic analysis showed that both the risk score and histological subtype were independent prognostic factors (Figs. 4B–4C). A nomogram containing the risk scores and histological subtype was constructed using the TCGA-STAD dataset (Fig. 4D). Based on this nomogram model, a calibration curve was plotted, showing a slope close to 1, indicating strong predictive accuracy of the model for the survival probability of patients with GC (Fig. 4E).

### Immune cells and immune checkpoints are linked to the risk score

**Figure 5:**
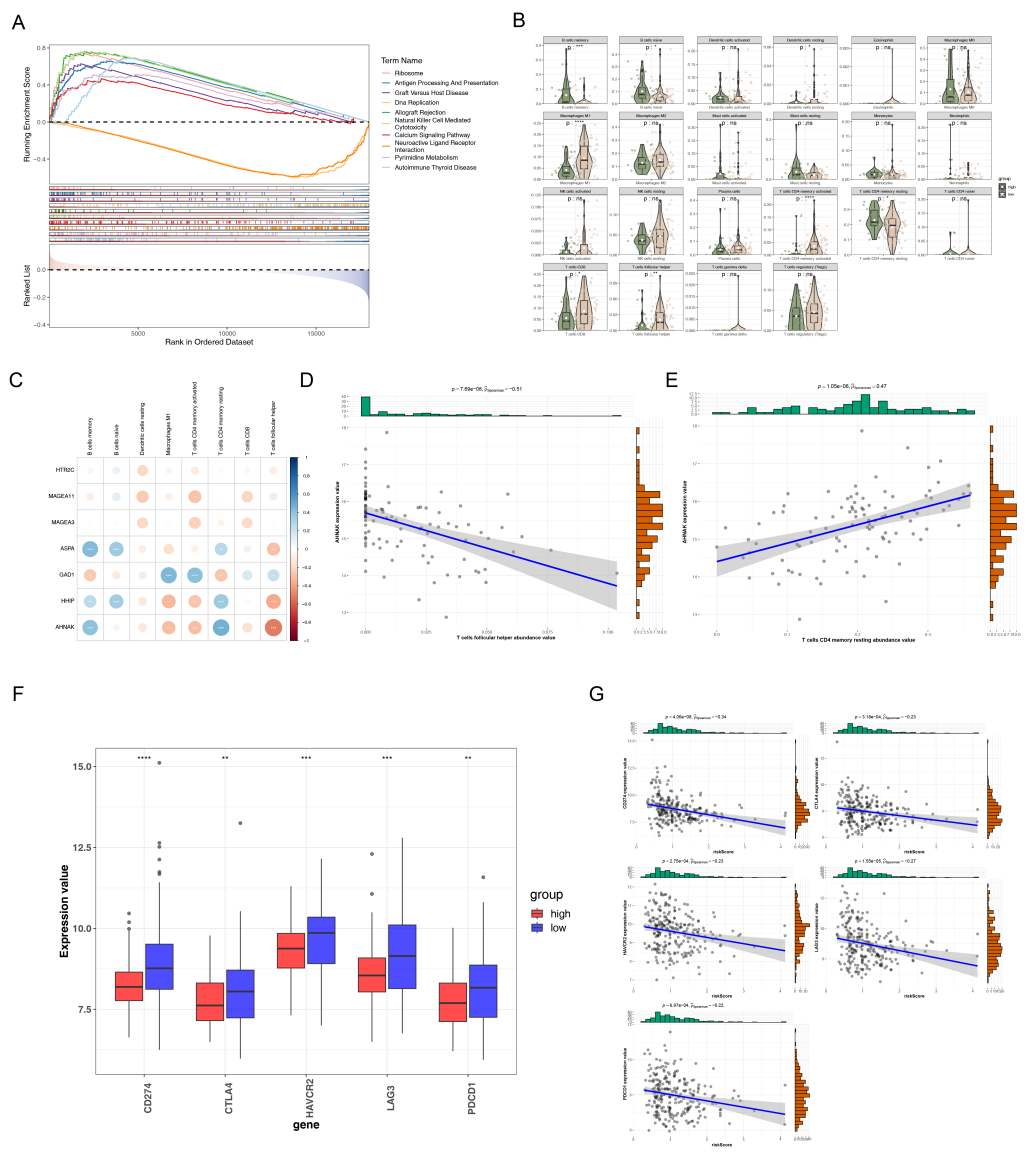
Immune infiltration and immune checkpoint analysis in high- and low-risk groups. (A) GSEA enrichment plots showing pathways enriched in the two risk groups (padj < 0.05). (B) Violin plots comparing the abundance of 22 immune cell types between the HRG and LRG. *p < 0.05, **p < 0.01, ***p < 0.001, ****p < 0.0001, ns: not significant; Wilcoxon rank-sum test. (C) Heatmap for the correlations between seven prognostic genes and eight differential immune cell types. (D) Scatter plot showing the highest positive correlation: *AHNAK vs.* resting CD4 memory T cells (*r* = 0.47, *p* < 0.001). (E) Scatter plot showing the highest negative correlation: *AHNAK vs.* T follicular helper cells (Tfh) (*r* = −0.51, *p* < 0.001). (F) Box plots comparing expression levels of eight immune checkpoints between HRG and LRG. **p < 0.01, ***p < 0.001, ****p < 0.0001; Wilcoxon rank-sum test. (G) Correlation analysis between immune checkpoint expression and risk score. CD274 exhibited the strongest negative correlation (*r* = −0.34, *p* < 0.001).

In the training set, DEGs between the two risk cohorts were primarily enriched in pathways such as ribosome, DNA replication, and pyrimidine metabolism (Fig. 5A). To determine the infiltration of immune cells in HRG and LRG, a heatmap was generated based on the training set to visualize the relative infiltration of 22 immune cell categories in 97 specimens (*p*-value < 0.05, Wilcoxon rank-sum test; Fig. S3). Significant differences (*p*-value < 0.05) were observed in eight immune cell types between the two groups. In the HRG, a higher infiltration of memory B cells and resting CD4 memory T cells was observed; however, lower infiltration was observed for resting dendritic cells, M1 macrophages, activated CD4 memory T cells, CD8 T cells, and T follicular helper cells (Tfh; Fig. 5B). A Spearman’s rank correlation analysis revealed associations between prognostic gene expression and immune cell infiltration. Of these, *AHNAK* exhibited a negative correlation with Tfh (*r* = −0.51, *p* < 0.001) and a positive correlation with resting CD4 memory T cells (*r* = 0.47, *p* < 0.001) (Figs. 5C–5E). In addition, analysis of immune checkpoint expression using the Wilcoxon rank-sum test revealed that *CD274*, *CTLA4*, *HAVCR2*, *LAG3*, and *PDCD1* were expressed at higher levels in the LRG (*p* < 0.05; Fig. 5F). A correlation analysis revealed that immune checkpoint expression levels were negatively correlated with risk scores, with CD274 showing the highest correlation among the checkpoints examined (*r* = −0.34, *p* < 0.05; Fig. 5G). These results suggest an association between risk score and immune checkpoint regulation, which warrants further study.

### Integrated regulatory network analysis reveals regulation in prognostic genes

**Figure 6:**
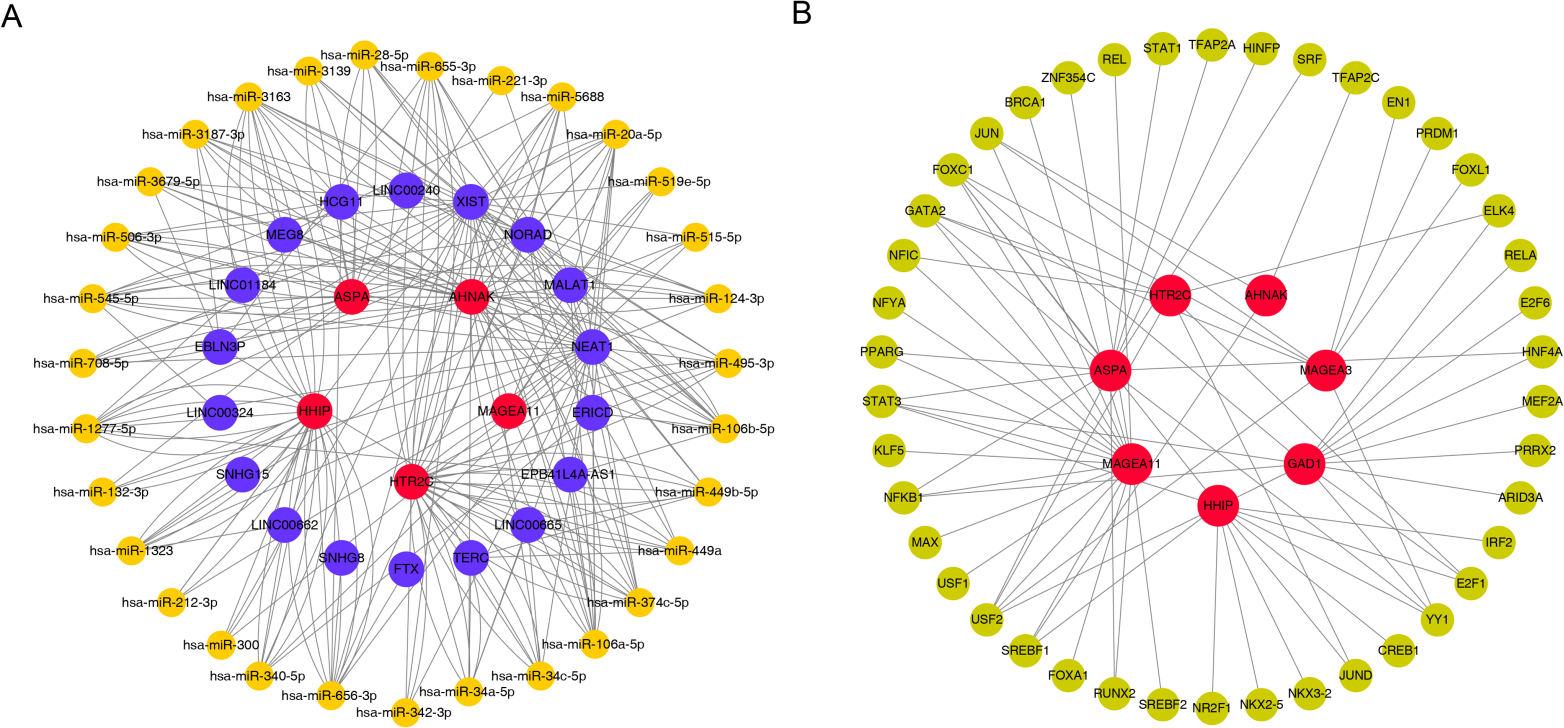
Predicted regulatory networks of the prognostic genes. (A) ceRNA network showing the potential interactions among mRNAs (red), lncRNAs (purple), and miRNAs (yellow). EPB41L4A-AS1 and ERICD may regulate *AHNAK* through hsa-miR-106a-5p. (B) TF–mRNA regulatory network. Red nodes represent mRNAs; yellow-green nodes represent TFs. *FOXC1* is predicted to co-regulate *MAGEA11*, *HHIP*, *MAGEA3*, *GAD1*, and *ASPA*.

A network consisting of 54 nodes and 238 edges was constructed based on the intersection of 65 shared miRNAs from the miRDB and MicroT-CDS databases, as well as 119 shared miRNA–lncRNA interaction pairs from the Starbase and miRNet databases. Based on the predictions, EPB41L4A-AS1 and ERICD were identified as potential upstream regulators of *AHNAK* through hsa-miR-106a-5p (Fig. 6A, Table S2). Similarly, TF prediction analysis indicated that *MAGEA11*, *HHIP*, *MAGEA3*, *GAD1*, and *ASPA* may be co-regulated by *FOXC1* (Fig. 6B, Table S3). FOXC1 may function as a core upstream regulatory node, synergistically modulating multiple prognosis-related genes associated with the TRP channel. These results provide insights into the transcriptional regulatory mechanisms underlying GC.

### The expression levels of the prognostic genes

**Figure 7:**
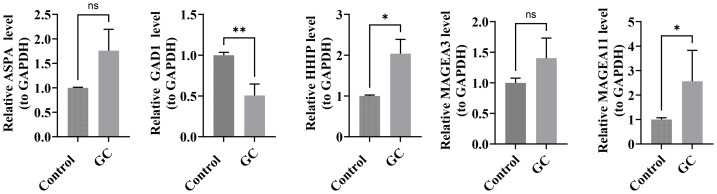
Preliminary RT-qPCR analysis of prognostic gene expression in clinical samples. Expression of *ASPA*, *MAGEA11*, *MAGEA3*, *GAD1*, and *HHIP* was measured in five paired GC (tumor) and adjacent normal tissues using RT-qPCR. *GAPDH* was used as an internal control. The data are presented as the mean ± SD (*n* = 5). *p < 0.05, **p < 0.01, ***p < 0.001; ns, not significant. *MAGEA11* expression was significantly higher in tumor tissues, consistent with the TCGA data. *MAGEA3* exhibited a trend toward higher expression. *ASPA*, *HHIP*, and *GAD1* showed expression trends opposite to the TCGA data, highlighting the need for validation in larger cohorts.

In the TCGA-STAD, the expression of *MAGEA11*, *MAGEA3*, and *GAD1* in the GC samples was significantly higher compared with those in the control samples, whereas *HHIP* and *ASPA* expression was significantly lower in the GC samples (Table S4). This prompted us to further use RT-qPCR techniques to validate the clinical expression of these prognostic genes in patients with GC. As shown in Fig. 7, RT-qPCR analysis of the five prognostic genes in five paired GC and adjacent normal tissues revealed heterogeneous expression patterns. Consistent with the TCGA-STAD dataset, *MAGEA11* expression was significantly higher in tumor tissues (*p* < 0.05). *MAGEA3* also showed a trend toward higher expression in the tumor samples, although it did not reach statistical significance, possibly because of the limited sample size and inter-individual variability. In contrast, the RT-qPCR results for *ASPA*, *HHIP*, and *GAD1* showed expression trends opposite to those observed in the TCGA-STAD dataset, whereas the differences for *HHIP* and *GAD1* reached statistical significance (*p* < 0.05). This discrepancy may be attributed to several factors, including the small sample size (*n* = 5), potential heterogeneity of GC tissues, differences in platform sensitivity between RNA-seq and RT-qPCR, and possible batch effects. These results highlight the need for validation in larger, independent cohorts to confirm the expression patterns of these genes.

## Discussion

TRP channels play a vital role in intracellular calcium regulation, and abnormal calcium signaling is associated with tumor development (Litan & Langhans, 2015; Murugan, Genautis & Voutsadakis, 2025). Studies have shown that TRP channels can affect tumor cell proliferation, invasion, and metastasis (Déliot & Constantin, 2015; Dhaouadi et al., 2023; Yue & Xu, 2021). Therefore, examining the relationship between these channels and GC is important for understanding the pathogenesis and treatment of GC. In the present study, seven prognostic genes related to GC (*HTR2C*, *MAGEA11*, *MAGEA3*, *ASPA*, *GAD1*, *HHIP*, and *AHNAK*) were identified based on univariate Cox regression and LASSO analyses. In addition, functional analysis, immune infiltration characterization, and regulatory network construction revealed potential biological processes and pathways associated with these genes, which provide insight for further mechanistic exploration.

The role of *HHIP* in GC remains controversial; however, the prevailing view is that it acts as a tumor suppressor. Silencing *HHIP* expression through high promoter methylation activates the Hedgehog signaling pathway, thereby promoting tumor progression. Conversely, overexpression of *HHIP* can inhibit tumor growth and metastasis (Akhtar et al., 2025; Song et al., 2020). Our data indicated that *HHIP* expression is downregulated in GC tissues (Table S4), which is consistent with the anticipated tumor-suppressive function described above. However, univariate Cox regression analysis revealed that *HHIP* is a prognostic risk factor. Its high expression is associated with poor patient prognosis. This appears to contradict descriptions of its tumor-suppressing role in the literature. Possible reasons for this include the function of *HHIP* may be dependent on tumor stage or subtype. Its prognostic significance may be independent of its classical regulatory role in the Hedgehog pathway. Therefore, the precise role of *HHIP* in GC and its value as a prognostic biomarker require further validation.

The melanoma antigen family A (MAGE-A) subfamily is abnormally expressed in a variety of malignant tumors and plays a role in tumor initiation and progression (Verma et al., 2024). In particular, MAGE-A11 and MAGE-A3, as cancer-testis antigens, have been reported to be highly expressed in GC tissue and are closely associated with poor patient prognosis (Gan et al., 2024; Jin et al., 2021; Wang et al., 2024). Mechanistic studies have shown that MAGE-A3 creates an immunosuppressive microenvironment through mechanisms, such as inhibiting CD8^+^ T-cell infiltration and promoting M2 macrophage polarization (Gan et al., 2024). Mechanistic studies indicate that MAGE-A3 shapes an immunosuppressive microenvironment through mechanisms, such as inhibiting CD8^+^ T-cell infiltration and promoting M2 macrophage polarization (Yu et al., 2022). Although MAGE-A3 is a potential target for immunotherapies, such as vaccines and Chimeric Antigen Receptor T-cell (CAR-T) therapy (Meng et al., 2021; Morgan et al., 2013), phase III clinical trials, such as the MAGRIT study in non-small cell lung cancer (NSCLC), failed to demonstrate a survival benefit, suggesting that clinical translation still faces challenges, such as patient heterogeneity and an insufficient immune response. In the present study, MAGE-A11 and MAGE-A3 were identified as genes associated with GC prognosis, which is consistent with their potential roles described in the aforementioned literature. It should be emphasized that this study only reveals a statistical association between these two genes and prognosis. Whether they are involved in GC progression through the aforementioned mechanisms remains to be determined.

The *HTR2C* gene demonstrates complex associations with tumor prognosis through metabolic and regulatory pathways. Its variants are linked to obesity and metabolic dysregulation (*e.g.*, hyperglycemia), which are known risk factors for tumor progression and chemotherapy resistance (He et al., 2022b). Mechanistically, *HTR2C* depletion activates the AKT/GSK3β/β-catenin signaling pathway, which is implicated in tumor proliferation and metastasis (Liu et al., 2024). Although direct prognostic studies in cancers are limited, *HTR2C* variants may serve as indirect prognostic markers by modulating host metabolic fitness and therapy responses (He et al., 2022b; Shen et al., 2025).

The *ASPA* gene has significant prognostic implications in various cancers. In PCa, decreased *ASPA* expression independently correlates with poorer patient prognosis (hazard ratio = 0.60, *P* = 0.018). It suppresses tumor proliferation, migration, and invasion by inhibiting LYN phosphorylation and downstream JNK1/2/C-Jun signaling (Weng et al., 2023). In HCC, *ASPA* inhibited tumor growth, enhanced chemosensitivity, and inactivated the MEKK1/NF-*κ*B signaling pathway in a xenograft model (Li & Qiu, 2024). In another study, GC exhibited reduced *ASPA* levels, which were associated with advanced disease and worse survival, whereas *ASPA* knockdown promoted proliferation and invasion (Han et al., 2023). Mechanistically, *ASPA* exerts enzyme-independent tumor-suppressive effects, suggesting its potential as a therapeutic target and prognostic biomarker.

*AHNAK* is a large scaffold protein that exhibits dual roles in tumor progression and prognosis in various cancers. In nasopharyngeal carcinoma, reduced *AHNAK* expression correlates with poor overall survival and promotes metastasis by upregulating annexin A2, suggesting its tumor-suppressive role (Lu et al., 2024). Conversely, *AHNAK2* overexpression in pancreatic ductal adenocarcinoma and lung adenocarcinoma (LUAD) is associated with advanced tumor stage, poor prognosis, and resistance to gemcitabine, which drives cell proliferation through KRAS/p53 and HGF/c-MET pathways (Chen et al., 2024; Li et al., 2023). *AHNAK* also modulates immune infiltration (*e.g.*, B cells, macrophages) in pancreatic cancer, thus affecting therapeutic outcomes (Ou et al., 2024). Interestingly, *AHNAK*/*AHNAK2* mutations may define a cancer subtype with enhanced immunogenicity and improved immunotherapy responses (Mai et al., 2024). These findings highlight the context-dependent prognostic and therapeutic implications of *AHNAK*.

The tumor immune microenvironment plays a significant role in the development and progression of GC. In the present study, we found that patients in the HRG experienced an immunosuppressive state, characterized by reduced Tfh infiltration and an increase in resting CD4^+^ memory T cells. Notably, *AHNAK* exhibited the strongest negative correlation with Tfh and the strongest positive correlation with resting CD4^+^ memory T cells. Tfh cells are important for germinal center formation and antibody production. Their accumulation in the tumor microenvironment is typically associated with a favorable prognosis and response to immunotherapy (Fujioka et al., 2026; Zhao et al., 2022). Conversely, an increased abundance of resting CD4^+^ memory T cells is associated with a poor prognosis (Ning et al., 2020). Thus, *AHNAK* may affect the immune microenvironment in GC by inhibiting Tfh differentiation or recruitment, or by altering the functional state of CD4^+^ T cells. Moreover, we found that the expression of immune checkpoints, such as CD274 and CTLA-4, was lower in the HRG, with CD274 showing a significant negative correlation with risk score. Combined with the current clinical reality of limited efficacy of immune checkpoint inhibitors in advanced GC (Kang et al., 2017; Kang et al., 2022). These results suggest that high-risk patients may respond poorly to immune checkpoint inhibitor therapy, which provides a basis for the development of personalized immunotherapy regimens in clinical practice.

To examine the upstream regulatory pathways of prognostic genes, we constructed ceRNA and TF regulatory networks. A network analysis predicted that the lncRNAs, EPB41L4A-AS1 and ERICD, may reverse the translational repression of *AHNAK* mRNA by competitively binding to hsa-miR-106a-5p. Previous studies have shown that EPB41L4A-AS1 acts as a tumor suppressor in various tumors, and its downregulation is associated with poor patient prognosis (Li et al., 2025b). Combined with our immune-related analyses, we hypothesized that in GC, the EPB41L4A-AS1/ERICD downregulation releases hsa-miR-106a-5p, thereby inhibiting *AHNAK* translation. Changes in *AHNAK* protein levels may affect Tfh differentiation through an unknown mechanism, ultimately promoting immune evasion and disease progression. In addition, transcriptional factor prediction analyses suggest that FOXC1 may co-regulate five genes, including *MAGEA11* and *HHIP*. Previous studies have indicated that FOXC1 is a key driver of malignant progression in various tumors and is closely associated with GC staging and poor prognosis (Sun et al., 2022). This provides insight for further studies into the transcriptional regulatory mechanisms of prognostic genes.

In the present study, we used transcriptomic data from TCGA-STAD and computational approaches to identify TRP channel-associated prognostic genes in GC, including *HTR2C*, *MAGEA11*, *MAGEA3*, *ASPA*, *GAD1*, *HHIP*, and *AHNAK*. GSEA, immune profiling, and regulatory network analyses suggest associations between these genes and immune cell infiltration as well as complex regulatory mechanisms, which may contribute to patient prognosis. These results provide potential biomarkers for informing personalized therapeutic strategies and predicting clinical outcomes.However, this study has several limitations. First, the significant disparity in the number of tumor and normal samples in the TCGA-STAD dataset may introduce bias into the initial differential expression analysis, potentially amplifying or masking true biological differences. Although we applied strict statistical thresholds to mitigate this effect, such sample imbalance remains an inherent limitation of public datasets. Therefore, future studies should validate the findings of this study using datasets with more balanced control samples. Second, this study has not yet been fully validated in a clinical setting, and the potential regulatory pathways and signaling networks require further exploration. Follow-up studies should elucidate the relevant regulatory mechanisms through larger clinical cohorts and functional experiments, providing stronger evidence for the models and targets proposed in this study.

## Conclusions

In this study, seven prognostic genes (*HTR2C*, *MAGEA11*, *MAGEA3*, *ASPA*, *GAD1*, *HHIP*, and *AHNAK*) related to TRP channels were identified in GC. This provides insight into the prognostic mechanisms of GC and offers a basis for further evaluation of personalized treatment strategies; however, the clinical utility of this model requires further validation in larger, prospective cohorts.

## Supplemental Information

10.7717/peerj.21468/supp-1 Supplemental Information 1 Raw data and code Original code for this study and raw data for PCR.

10.7717/peerj.21468/supp-2 Supplemental Information 2 Identification of DEGs in GC and candidate gene enrichment analysis (A) Heatmap of the differentially expressed genes. Red indicates high expression, and purple indicates low expression. (B) Cluster dendrogram of co-expression modules identified by WGCNA. (C) Heatmap for the top differentially expressed genes between high- and low-ssGSEA score groups. (D) Bar plot of GO enrichment analysis for candidate genes (padj < 0.05). (E) Bar plot of KEGG pathway enrichment analysis for candidate genes (padj < 0.05).

10.7717/peerj.21468/supp-3 Supplemental Information 3 Performance evaluation of the risk model (A) Performance of the risk model in the TCGA-STAD training set (*n* = 244). (A-1) Risk score distribution and survival status of patients . Higher risk scores were associated with increased mortality. (A-2) Kaplan–Meier survival curves for the high-risk group (HRG) and low-risk group (LRG) (log-rank test, *p* < 0.0001). (A-3) Time-dependent ROC curves for 1-, 3-, and 5-year survival prediction. The AUC values were all greater than 0.6, indicating the moderate predictive accuracy of the risk model. (B) Internal validation set (*n* = 104). (B-1) Risk score distribution and survival status. (B-2) Kaplan–Meier survival curves (log-rank test, *p* < 0.05). (B-3) Time-dependent ROC curves for 1-, 3-, and 5-year survival prediction.

10.7717/peerj.21468/supp-4 Supplemental Information 4 Stacked bar plot showing the relative proportions of 22 immune cell types in each sample (CIBERSORT, *p* < 0.05)

10.7717/peerj.21468/supp-5 Supplemental Information 5 PCR primer sequences The primer sequences for MAGEA11, MAGEA3, ASPA, GAD1, and HHIP are shown in Table S1, with GAPDH used as the internal reference gene.

10.7717/peerj.21468/supp-6 Supplemental Information 6 The network of lncRNA-miRNA-mRNA interactions

10.7717/peerj.21468/supp-7 Supplemental Information 7 Prognostic genes regulated by FOXC1

10.7717/peerj.21468/supp-8 Supplemental Information 8 The expression differences of prognostic genes between GC and control samples in TCGA-STAD cohort

10.7717/peerj.21468/supp-9 Supplemental Information 9 MIQE checklist

## Additional Information and Declarations

### Data Availability

The following information was supplied regarding data availability: The raw data and code are available in the Supplemental Files. The data used in this study are available from the University of California, Santa Cruz (UCSC) Xena database: - https://xenabrowser.net/datapages/?cohort=GDC%20TCGA%20Stomach%20Cancer%20(STAD)&removeHub=https%3A%2F%2Fxena.treehouse.gi.ucsc.edu%3A443
The raw sequence data are available at NCBI: GSE84433.
